# Microsomal triglyceride transfer protein contributes to lipid droplet maturation in adipocytes

**DOI:** 10.1371/journal.pone.0181046

**Published:** 2017-08-09

**Authors:** Larry L. Swift, Joseph D. Love, Carla M. Harris, Benny H. Chang, W. Gray Jerome

**Affiliations:** 1 Department of Pathology, Microbiology and Immunology, Vanderbilt University School of Medicine, Nashville, Tennessee, United States of America; 2 Research Service, Veterans Affairs, Tennessee Valley Health Care System, Nashville, Tennessee, United States of America; 3 Departments of Molecular & Cellular Biology and Medicine, Baylor College of Medicine, Houston, Texas, United States of America; Fundacao Oswaldo Cruz, BRAZIL

## Abstract

Previous studies in our laboratory have established the presence of MTP in both white and brown adipose tissue in mice as well as in 3T3-L1 cells. Additional studies demonstrated an increase in MTP levels as 3T3-L1 cells differentiate into adipocytes concurrent with the movement of MTP from the juxtanuclear region of the cell to the surface of lipid droplets. This suggested a role for MTP in lipid droplet biogenesis and/or maturation. To probe the role of MTP in adipocytes, we used a *Cre*-Lox approach with *aP2-Cre* and *Adipoq-Cre* recombinase transgenic mice to knock down MTP expression in brown and white fat of mice. MTP expression was reduced approximately 55% in white fat and 65–80% in brown fat. Reducing MTP expression in adipose tissue had no effect on weight gain or body composition, whether the mice were fed a regular rodent or high fat diet. In addition, serum lipids and unesterified fatty acid levels were not altered in the knockdown mice. Importantly, decreased MTP expression in adipose tissue was associated with smaller lipid droplets in brown fat and smaller adipocytes in white fat. These results combined with our previous studies showing MTP lipid transfer activity is not necessary for lipid droplet initiation or growth in the early stages of differentiation, suggest that a structural feature of the MTP protein is important in lipid droplet maturation. We conclude that MTP protein plays a critical role in lipid droplet maturation, but does not regulate total body fat accumulation.

## Introduction

Our laboratory was the first to report the presence of microsomal triglyceride transfer protein (MTP) in adipocytes of mice, rats, and humans [1]. Immunohistochemical studies revealed MTP surrounding lipid droplets in both brown and white fat. Immunofluorescence studies suggested that MTP was associated with the surface of the lipid droplet [1], an observation that was confirmed by confocal and electron microscopic studies [2]. In addition, we showed that MTP was expressed in mouse 3T3-L1 cells [1] and that protein levels increased nearly five-fold as the cells differentiated into adipocytes [2]. Furthermore, as the cells differentiated, MTP moved from the juxtanuclear region of the cell to the surface of the droplet [2]. Taken together, our observations suggested that MTP is involved in lipid droplet biology. In particular, we hypothesized that MTP was involved in the formation and/or maturation of lipid droplets. However, our studies with 3T3-L1 cells have shown that inhibition of MTP activity has no effect on the differentiation of these cells into adipocytes as assessed by the percent of cells that contain lipid droplets or the number of lipid droplets per cell [2]. In addition, inhibition of MTP activity had no effect on the movement of triglyceride out of fully differentiated 3T3-L1 cells either as a lipid complex or via lipolysis [2]. While these are important observations, they provide only limited insight into the primary function of MTP within the adipocyte.

Recently, Bakillah and Hussain [3] reported that *aP2-Cre* mediated ablation of the *Mttp* gene in adipose tissue of mice resulted in resistance to high fat diet-induced obesity. The adipose tissue-specific MTP knockdown (A-*Mttp*^-/-^) mice had reduced white adipose tissue with increased numbers of smaller size adipocytes compared with control mice. In addition, they reported that these knockdown mice were also protected from high-fat diet-induced fatty liver. The authors suggested that inactivation of MTP in adipose tissue might be useful as an “anti-obesity drug”.

Our laboratory has also used the *Cre*-Lox approach to reduce expression of MTP in adipose tissue in mice. We found that suppression of MTP expression in adipose tissue using mice expressing *Cre*-recombinase under the control of the *aP2* promoter or the *Adipoq* promoter did not result in resistance to high fat diet-induced obesity nor did it protect the animal from high fat diet-induced fatty liver. It did, however, significantly influence the size of white adipocytes and the size distribution of lipid droplets within brown fat. Thus, we are in agreement with Bakillah and Hussain’s conclusions that MTP plays an important role in the maturation of lipid droplets; however, based on our results, we feel the precise role of MTP in controlling accretion of body fat seems variable, and thus this aspect of MTP biology in adipose tissue remains a topic requiring additional research.

## Materials and methods

### Antibody and reagents

Rabbit anti-MTP was developed in our laboratory and has been described previously [4, 5]. Goat anti-rabbit IgG conjugated to horseradish peroxidase was purchased from Promega (Madison, WI).

### Animals and diets

Transgenic mice expressing *Cre* recombinase under the control of the mouse *Fabp4*, fatty acid binding protein 4, adipocyte promoter (B6.Cg-Tg(Fabp4-cre)1Rev/J;Jackson) also known as *aP2*-*Cre* mice, and mice expressing *Cre*-recombinase under the control of the mouse adiponectin (*Adipoq*) promoter (B6;FVB-Tg(adipoq-cre)1Evdr/J), also known as *Adipoq*-*Cre* mice, were purchased from The Jackson Laboratory. Mice in which exons 5 and 6 of the *Mttp* gene are flanked by *loxP* sites were developed by Chang *et al*. [6] Mice, homozygous for the floxed *Mttp* allele (f^+/+^) were mated with *aP2*-*Cre* or *Adipoq*-*Cre* mice to generate f^+/+^;*Cre* mice. Since the *loxP* sites surround exons 5 and 6, these mice should be deficient in both MTP-A and MTP-B in targeted tissues.

Mice were genotyped at 4 weeks of age, and age-matched male mice (f^+/+^, f^+/-^, and f^+/+^;*Cre*) from *aP2* and *Adipoq Cre* matings were maintained on regular diet (PicoLab Laboratory Rodent Diet 5L0D, LabDiet, St. Louis, MO) for as long as 29 weeks. In addition, groups of mice (f^+/+^, f^+/-^, and f^+/+^;*Cre* from *aP2* and *Adipoq Cre* matings) were fed the regular rodent diet for 14 weeks and then placed on a semi purified high fat diet (Harlan Teklad TD.88137, 42.0, 42.7 and 15.2% of kcal/g as fat, carbohydrate, and protein, respectively) for 8 weeks. In one set of experiments, f^+/+^, f^+/-^;*Cre* and f^+/+^;*Cre* mice from *aP2* crosses were placed on the high fat diet at weaning and fed the diet for 24 weeks. Mice were weighed weekly or biweekly, and body composition was determined using a Minispec Model mq7.5 pulsed NMR system (Bruker Instruments). Because we observed no differences between f^+/+^ and f^+/-^ mice in any of the measurements, we included both f^+/+^ and f^+/-^ mice in the control groups.

At the end of the dietary periods, the mice were anesthetized and blood was collected via the inferior vena cava. Gonadal fat, intrascapular brown fat, and liver were removed and weighed, and pieces were fixed in 10% formalin for histologic examination. The remainder of the tissue was frozen for further analysis.

### Sample preparation, SDS-polyacrylamide gel electrophoresis, and immunoblotting

Adipose tissue (~50 mg) was disrupted in ice-cold RIPA buffer (100–150 μl) using a glass homogenizer. The homogenate was transferred to an eppendorf tube and rotated at 4°C for 4–6 hours and spun at 13,000 rpm for at least 30 min. The middle layer was collected, and protein concentration was determined using the bicinchoninic acid (BCA) assay. Aliquots of the extract containing 20–40 μg protein were solubilized in NuPAGE LDS sample buffer, and the proteins were separated by SDS-PAGE using NuPAGE bis-tris gels (4–12% gradients) (Life Technologies) with morpholinepropanesulfonic acid SDS running buffer [7]. The proteins were transferred to nitrocellulose membranes. The membranes were blocked in TBS with 5% non-fat milk, incubated overnight at 4°C with primary antibody, washed extensively, and incubated for 1 h at room temperature with the appropriate secondary antibody conjugated with horseradish peroxidase. Bands were visualized using Western Lightning® Plus-ECL enhanced chemiluminescence substrate (Perkin Elmer, Waltham, MA) and quantitated using a Syngene G:Box Chemi XT4 System (Syngene, Frederick, MD).

### Serum and tissue lipid quantitation

Total serum cholesterol and triglycerides were measured by standard enzymatic assays utilizing kits from Cliniqa (Cliniqa Corp., San Marcos, CA). NEFA were quantitated using a commercial enzymatic assay (Wako Chem., Inc., Richmond, VA). Lipids were extracted from pieces of liver using the method of Folch-Lees [8]. Individual lipid classes were separated by thin layer chromatography using Silica Gel 60 A plates developed in petroleum ether, ethyl ether, acetic acid (80:20:1) and visualized by rhodamine 6G. The triglyceride band was scraped from the plates and methylated using BF3 /methanol (Sigma-Aldrich, St. Louis, MO) as described by Morrison and Smith [9]. The methylated fatty acids were extracted and analyzed by gas chromatography. Gas chromatographic analyses were carried out on an Agilent 7890A gas chromatograph equipped with flame ionization detectors and a capillary column (SP2380, 0.25 mm x 30 m, 0.25 μm film, Sigma-Aldrich). Helium was used as the carrier gas. The oven temperature was programmed from 160°C to 230°C at 4°C/min. Fatty acid methyl esters were identified by comparing the retention times to those of known standards. Inclusion of trieicosenoin (C20:1ω9) in the extraction procedure permitted quantitation of the tissue triglyceride.

### Histochemical analysis

Tissues were prepared for histological analysis by the Translational Pathology Shared Resource at Vanderbilt. Formalin-fixed tissues were embedded in paraffin, sectioned (5 μm) and stained with hematoxylin and eosin. Images were captured using an unbiased, hierarchical sampling scheme. For each image, a subset of droplets (>100 per animal) was selected for measurement using a sampling grid, which randomly selected the droplets and the angle at which the diameter was measured.

### Ethics statement

This study was carried out in strict accordance with the recommendations in the Guide for the Care and Use of Laboratory Animals of the National Institutes of Health. The protocol was approved by the Institutional Animal Care and Use Committee (IACUC) of Vanderbilt University (Protocol Number: V/14/025).

## Results

### MTP expression in adipose tissue of mice

**Fig 1:**
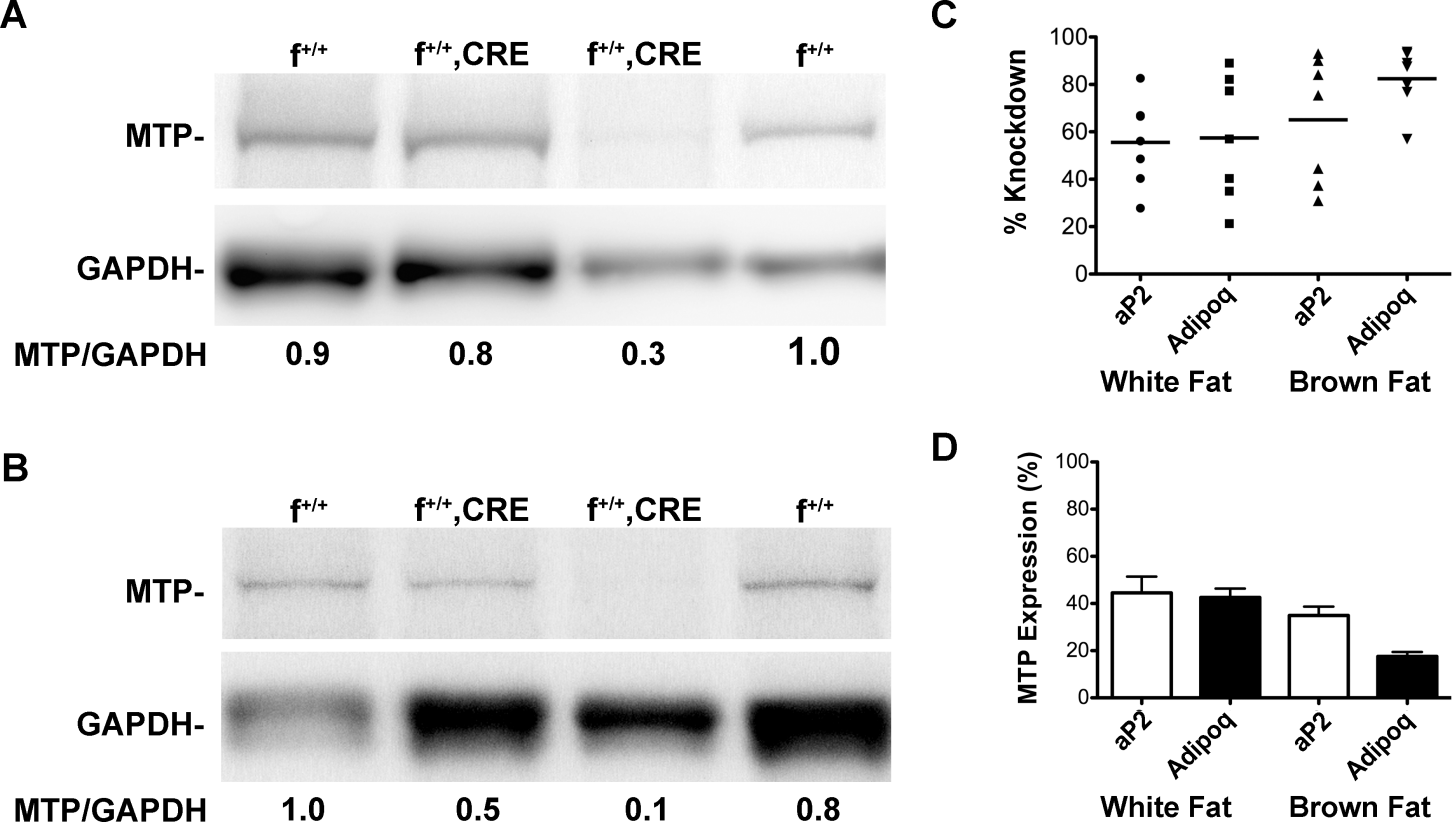
Variability of MTP knockdowns within and between litters. (A,B) Immunoblots showing MTP expression in white (A) and brown (B) fat from male littermates derived with the *AdipoQ* transgene. Note the differences in the effectiveness of the MTP knockdown in the two f^+/+^;*Cre* mice in both white and brown fat. (C) Scatter plots showing relative knockdown of MTP in white and brown fat from *aP2* and *Adipoq* crosses (n = 7/group). (D) Data from C showing average MTP expression in white and brown fat of knockdown mice (mean ± s.e.m., n = 7).

Mice in which *loxP* sites had been inserted around exons 5 and 6 were crossed with mice expressing *Cre*-recombinase under the control of the *aP2* (*FABP4*) promoter as well as with mice expressing *Cre*-recombinase under the control of the *Adipoq* (adiponectin) promoter. MTP protein expression in both white and brown fat was monitored by SDS-PAGE. The effectiveness of the *Cre*-lox approach to suppress MTP expression was highly variable between litters as well as within litters. Fig 1A and 1B show MTP protein expression in white and brown fat, respectively, from four male littermates, two of which were f^+/+^;*Cre* mice. In one f^+/+^;*Cre* mouse MTP expression was reduced ~15% in white fat and ~45% in brown fat. In the second f^+/+^;*Cre* mouse MTP expression was reduced ~70% in white fat and ~90% in brown fat. Because all the f^+/+^;*Cre* mice expressed some level of MTP in adipose tissue, we will refer to them as knockdown mice, not knockout mice. The variability of the knockdown across litters is shown in Fig 1C. The average knockdown of MTP in white fat from *aP2* or *Adipoq* mice was ~55% (55.5 ± 7.0, n = 7, vs. 57.4 ± 9.9, n = 7, respectively). Knockdown of MTP was more pronounced in brown fat, averaging 65% (65.1 ± 9.9, n = 7) in *aP2* mice and 82.4% (82.4 ± 4.9, n = 7) in *Adipoq* mice. Thus, in white fat MTP expression was approximately 45% of control for each of the *Cre* transgenes (Fig 1D); for brown fat MTP expression was approximately 35% of control levels in the *aP2* mice and 18% of control in the *Adipoq* mice (Fig 1D).

### Weight gain

**Fig 2:**
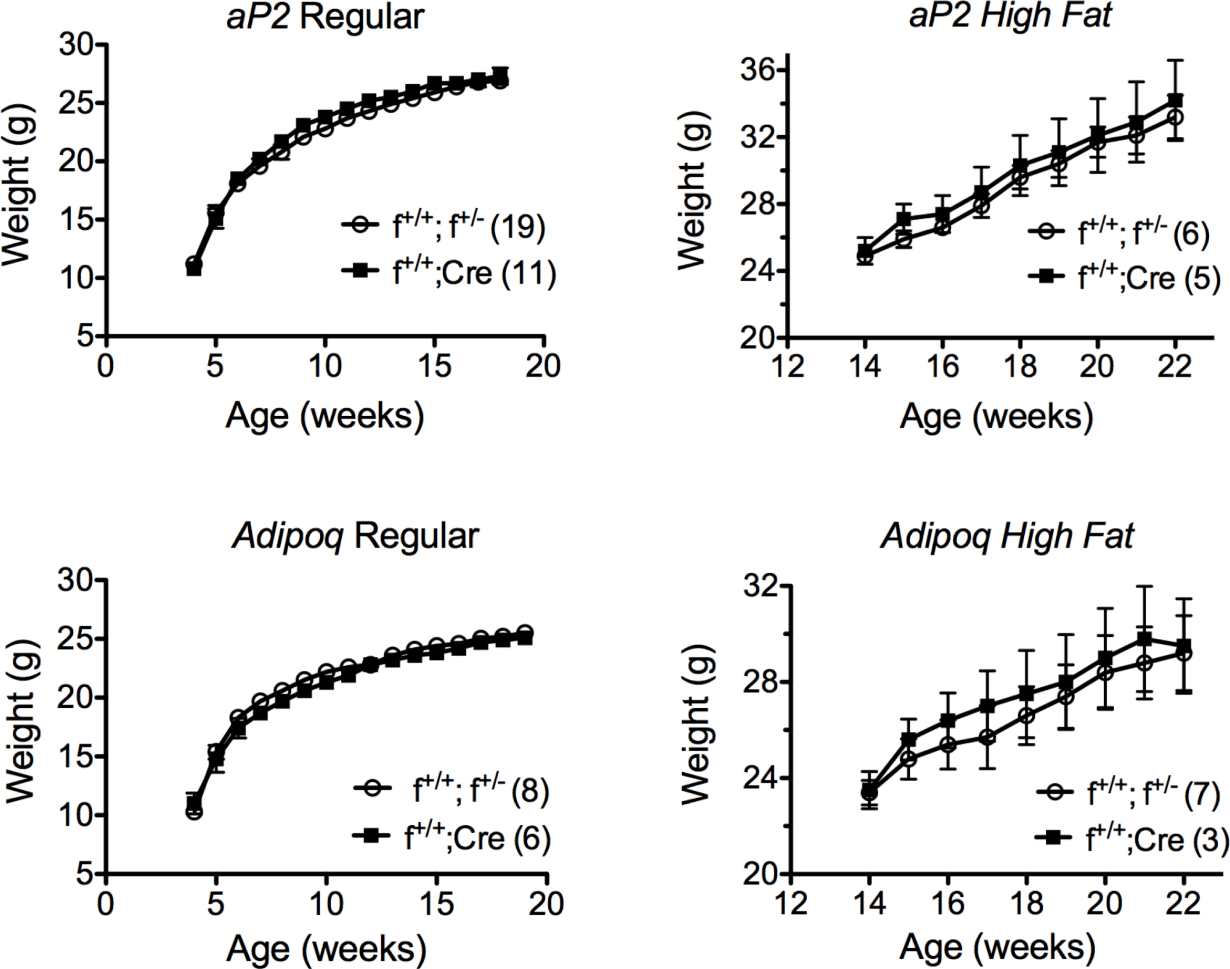
Weight gain of control (f^+/+^, f^+/-^) and MTP knockdown (f^+/+^;*Cre*) mice on regular rodent or high-fat diet. Male mice from either *aP2* or *Adipoq* crosses were placed on the regular diet at weaning and weighed weekly for 14–15 weeks. Mice fed the high-fat diet were maintained on the regular diet until 14 weeks of age at which time they were switched to the high-fat diet. Body weight and composition were determined weekly. Data, mean ± s.e.m.

**Fig 3:**
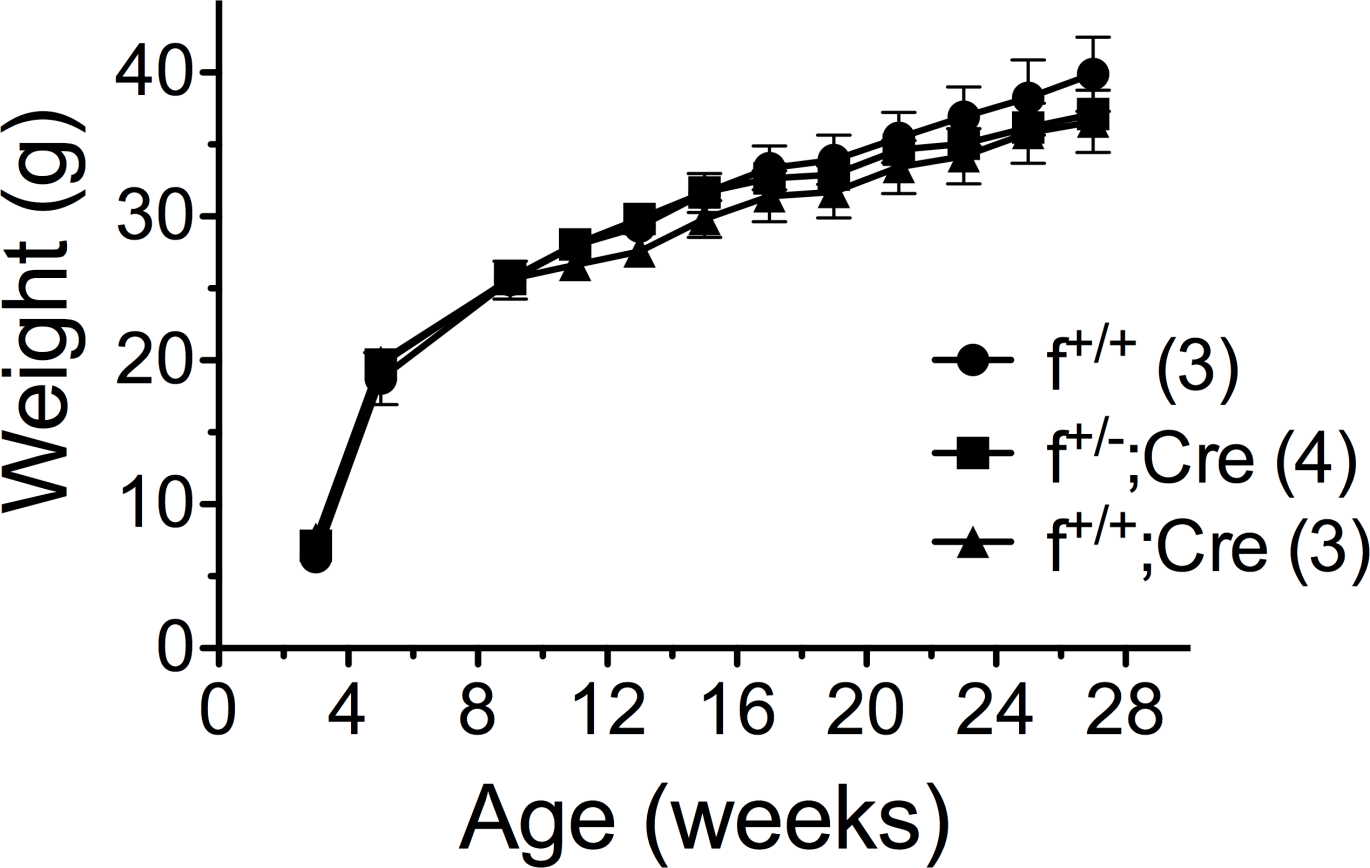
Effect of high fat diet on weight gain in control and knockdown mice. Male mice from *aP2* crosses were fed the high-fat diet from weaning (4 weeks) until 28 weeks of age. Mice were weighed bi-weekly. Numbers in parentheses represent the number of mice in each group. Data, mean ± s.e.m.

Knockdown (f^+/+^;*Cre*) and control (f^+/+^, f^+/-^) mice from *aP2* and *Adipoq* crosses maintained on regular rodent diet gained weight similarly (Fig 2). These studies were carried out for 19 weeks; however, we have extended some studies for as long as 29 weeks with the same results. Because the knockdown in our animals was variable and never 100% effective, we reasoned that residual MTP in fat might be sufficient to maintain normal adipocyte function and physiology under regular dietary conditions, but might not be sufficient under conditions of elevated fat intake and storage. Consequently, we fed mice the high-fat diet for 24 weeks beginning at weaning. Not seeing any significant differences in weight between the f^+/+^;*Cre* and control mice (Fig 3), we modified the feeding regimen instituting the high-fat diet when the mice were fully mature. In so doing we reasoned we could evaluate the full impact of the diet on fat storage, removing any differences between the two groups with regard to normal development of lean body mass. Mice were then maintained on regular rodent diet until 14 weeks of age, at which time they were placed on the high-fat diet for 8 weeks. The mice were weighed and body composition assessed weekly. We saw no differences in weight between knockdown and control animals from either the *aP2* or *Adipoq* crosses (Fig 2).

### Tissue analysis

At the end of the dietary periods, the mice were sacrificed, and tissues were collected and weighed. We saw no differences in weight of livers or the mass of gonadal fat or intrascapular brown fat between control and knockdown mice from *aP2* or *Adipoq* crosses on either regular or high-fat diet (Table 1). In addition, there were no differences in body composition between control and knockdown mice fed high fat diet for 8 weeks after 14 weeks on a regular rodent diet (Table 2) and no differences in serum lipid or unesterified fatty acid levels between control and knockdown mice on either regular rodent or high-fat diet (Table 3).

**Table 1:** Tissue weights from control and knockdown mice fed regular rodent diet or high fat diet*.

		Liver (g)		White Fat (mg)		Brown Fat (mg)	
*Cre*-Promoter	Genotype	Regular	High fat	Regular	High fat	Regular	High fat
*aP2*	f^+/+^; f^+/-^ (12,6)	1.36 ± 0.05	1.56 ± 0.10	354.0 ± 16.1	1687.8 ± 114.4	182.3 ± 8.3	540.8 ± 41.7
	f^+/+^;Cre (8,5)	1.38 ± 0.06	1.69 ± 0.15	341.3 ± 16.9	1663.6 ± 237.4	185.4 ± 9.5	553.6 ± 75.4
*Adipoq*	f^+/+^; f^+/-^ (5,7)	1.24 ± 0.04	1.25 ± 0.10	416.2 ± 30.7	1113.7 ± 138.8	197.8 ± 11.9	394.3 ± 47.8
	f^+/+^;Cre (4,3)	1.22 ± 0.03	1.34 ± 0.18	383.0 ± 21.0	1125.0 ± 331.7	174.0 ± 9.3	377.8 ± 105.5

*Mice fed the regular diet were 19 weeks old when euthanized and tissues were removed. High fat diet-fed mice were fed regular rodent diet until 14 weeks of age and then fed the high fat diet for 8 weeks. Data represent mean ± s.e.m. Numbers in parentheses represent number of mice in regular, high fat diet groups.

**Table 2:** Body composition of *aP2-Cre* and *Adipoq-Cre* mice fed a high fat diet*.

	*aP2-Cre*				*Adipoq-Cre*			
	0 Weeks		8 Weeks		0 Weeks		8 Weeks	
	f^+/+^; f^+/-^ (6)	f^+/+^;Cre (5)	f^+/+^; f^+/-^ (6)	f^+/+^;Cre (5)	f^+/+^; f^+/-^ (7)	f^+/+^;Cre (3)	f^+/+^; f^+/-^ (7)	f^+/+^;Cre (3)
Fat (g)	1.78 ± 0.15	1.75 ± 0.16	8.53 ± 0.67	8.90 ± 1.67	1.86 ± 0.13	2.10 ± 0.24	6.39 ± 1.04	6.46 ± 2.15
Muscle (g)	18.72 ± 0.31	18.82 ± 0.64	20.15 ± 0.60	20.39 ± 1.04	16.93 ± 0.37	16.89 ± 0.51	18.08 ± 0.49	18.33 ± 0.43
% Fat	7.17 ± 0.68	6.95 ± 0.53	25.55 ± 1.37	25.17 ± 3.52	7.91 ± 0.53	8.71 ± 0.83	21.14 ± 2.48	21.13 ± 6.09
% Muscle	75.08 ± 0.39	74.90 ± 0.53	60.86 ± 1.07	59.97 ± 1.88	71.60 ± 0.85	73.11 ± 0.96	62.51 ± 1.92	62.74 ± 4.79

*Mice were fed regular rodent diet until 14 weeks of age (0 weeks) followed by feeding the high fat diet for 8 weeks. Body composition was determined using a Minispec Model mq7.5 pulsed NMR system (Bruker Instruments). Data represent mean ± s.e.m. Numbers in parentheses represent number of mice in each group.

**Table 3:** Serum lipids of mice fed regular rodent or high fat diets*.

		Free Fatty Acids (mM)		Triglycerides (mg/dl)		Cholesterol (mg/dl)	
*Cre*-Promoter	Genotype	Regular	High fat	Regular	High fat	Regular	High fat
*aP2*	f^+/+^;f^+/-^ (8,5)	0.50 ± 0.05	0.82 ± 0.08	75.6 ± 6.8	63.0 ± 10.3	90.6 ± 2.8	198.8 ± 18.7
	f^+/+^;Cre (11,5)	0.54 ± 0.05	0.71 ± 0.10	63.7 ± 5.9	50.6 ± 9.5	82.5 ± 3.4	181.2 ± 15.5
*Adipoq*	f^+/+^;f^+/-^ (4,2)	0.97 ± 0.16	0.56 ± 0.04	56.8 ± 16.18	24.5 ± 2.1	80.8 ± 13.8	177.0 ± 7.1
	f^+/+^;Cre (3,4)	0.97 ± 0.20	0.82 ± 0.27	45.0 ± 24.1	33.3 ± 9.4	87.3 ± 17.0	160.7 ± 15.9

*Mice on regular diet were fed from weaning until 19 weeks of age. Mice on the high-fat diet regimen were fed regular rodent diet from weaning until 14 weeks followed by 8 weeks on the high fat diet. Data represent mean ± s.e.m. Numbers in parentheses represent number of mice in regular, high fat diet groups.

### Histology

**Fig 4:**
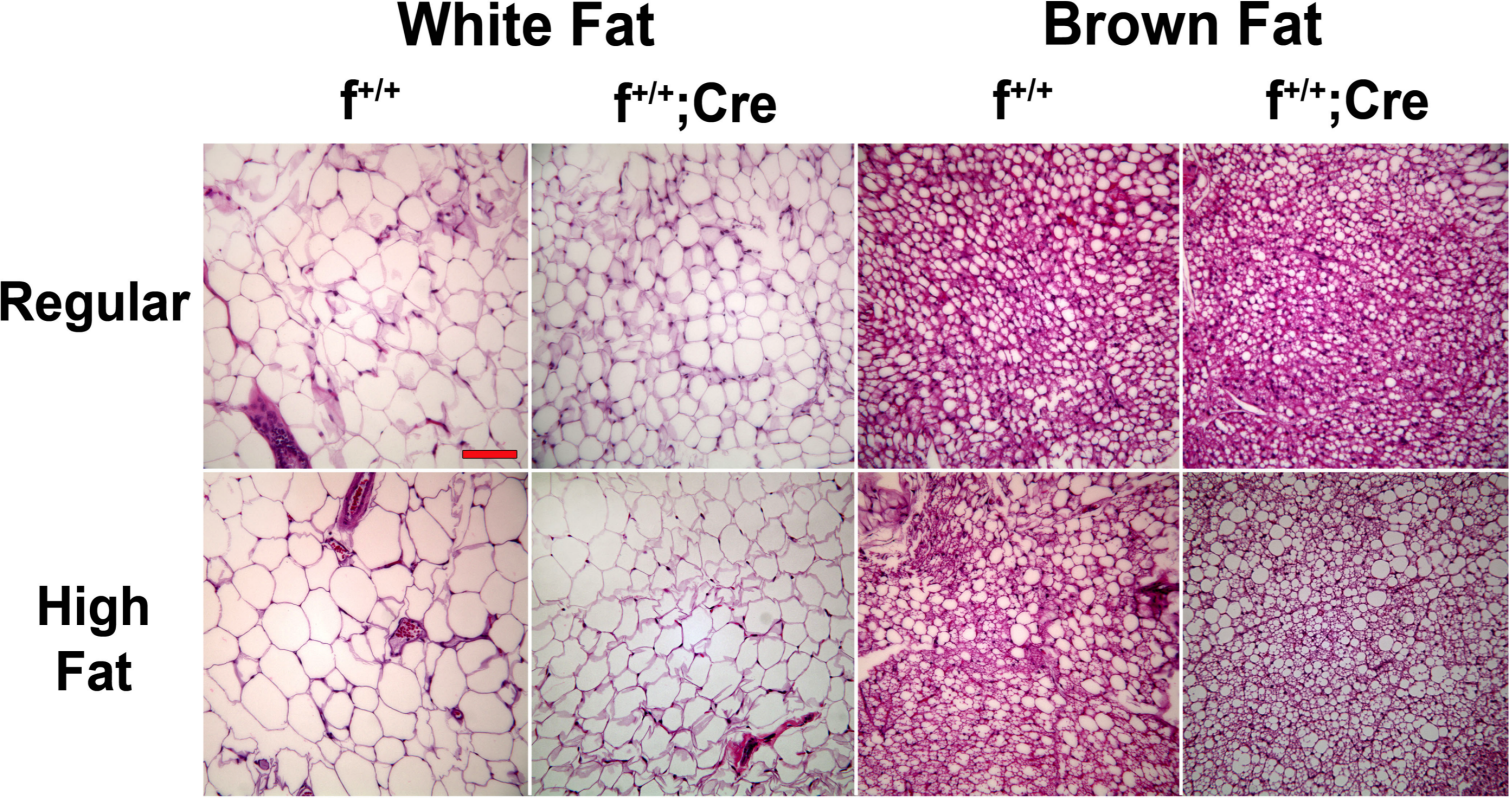
Microscopic analyses of white and brown fat from f^+/+^ and f^+/+^;*Cre* mice on regular and high-fat diets. Male mice from *Adipoq* crosses were fed a regular diet for 14 weeks and then either switched to high fat diet for another 8 weeks or maintained on the regular diet for 5 additional weeks. At the end of the dietary periods the mice were sacrificed, and pieces of white (gonadal) and brown fat were fixed, sectioned and stained with hematoxylin and eosin. Magnification bar = 100 μm.

**Fig 5:**
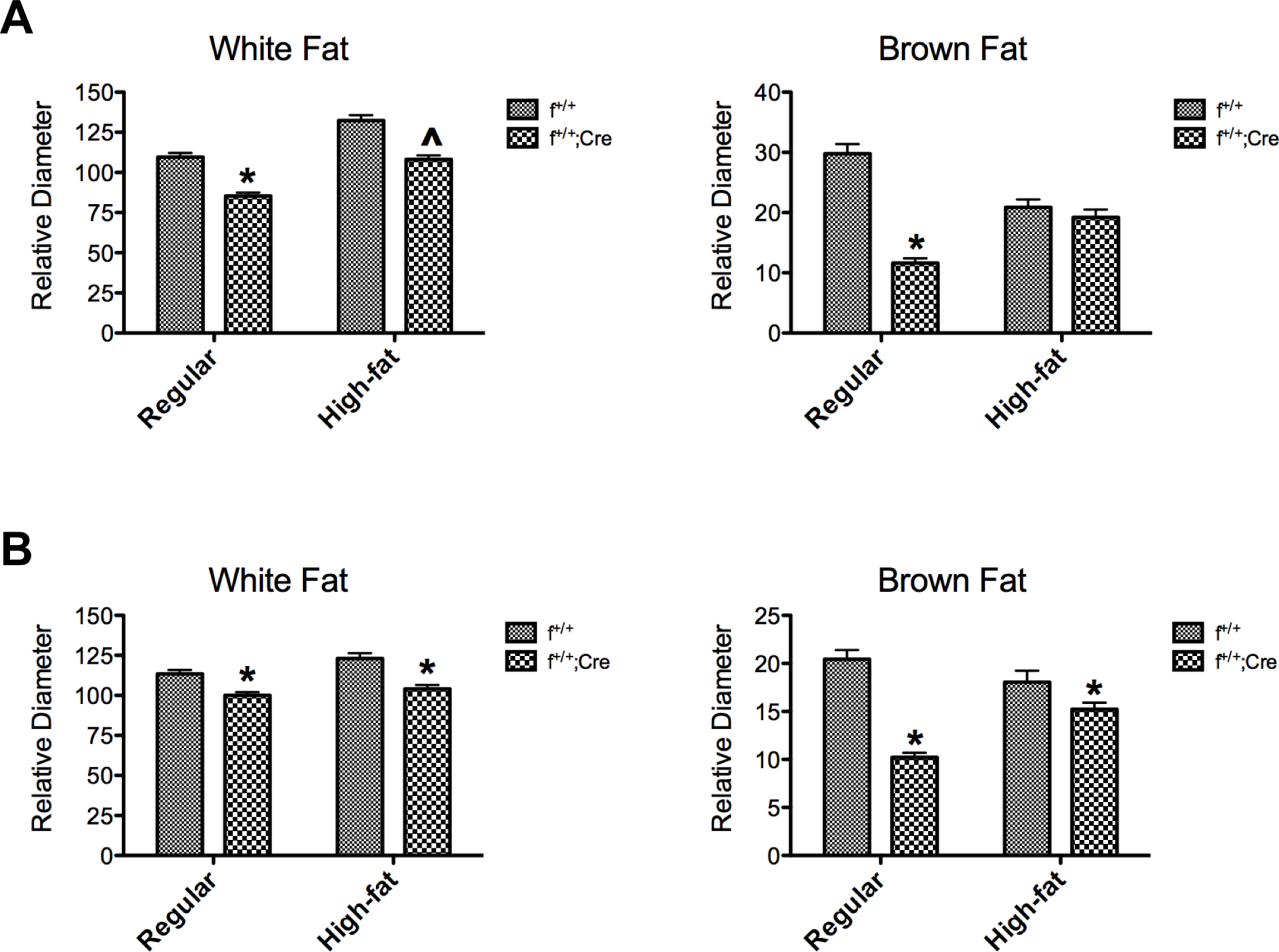
Adipocyte size in white fat and lipid droplet size in brown fat from f^+/+^ and f^+/+^;*Cre* mice. **C**ontrol and MTP knockdown mice derived from *Adipoq-Cre* (A) and *aP2-Cre* (B) crosses were fed a regular diet for 14 weeks and then either switched to high fat diet for another 8 weeks or maintained on the regular diet for 5 additional weeks. Pieces of white and brown fat were fixed, sectioned and stained with hematoxylin and eosin. Images were captured and droplets were sized using a sampling grid, which randomly selected the droplets and the angle at which the diameters were measured. * Significantly different from control, p < .0001; ^ Significantly different from control, p < .001.

Representative H&E sections of brown and white fat from control and knockdown mice from *aP2-Cre* and *Adipoq-Cre* crosses fed regular or high-fat diet are shown in Fig 4. Adipocytes in white fat from control and knockdown mice on regular rodent diet shared similar morphology with no major differences in structure; however, lipid droplets in white fat from knockdown mice were 5–25% smaller in diameter than those from the control mice (Fig 5). Likewise lipid droplets in brown fat from the knockdown mice were 50–60% smaller than droplets found in brown fat from control mice (Fig 5). The results from *Adipoq-Cre* (Fig 5A) and *aP2-Cre* (Fig 5B) crosses were nearly identical. In general the high fat diet led to an increase in the size of lipid droplets in white fat; however, the differences between control and knockdown mice were still apparent with lipid droplets in white fat from the knockdown mice averaging 15–20% smaller than in control mice (Fig 5). Interestingly, the effects of the high fat diet on lipid droplet size in brown fat were more complex. High fat diet led to a 10–30% decrease in lipid droplet size in control mice; however, it produced a 50–65% increase in lipid droplet diameter in the knockdown mice. Finally, we observed similar degrees of macrophage infiltration in control and knockdown mice whether the animals were on regular rodent diet or the high fat diet.

**Fig 6:**
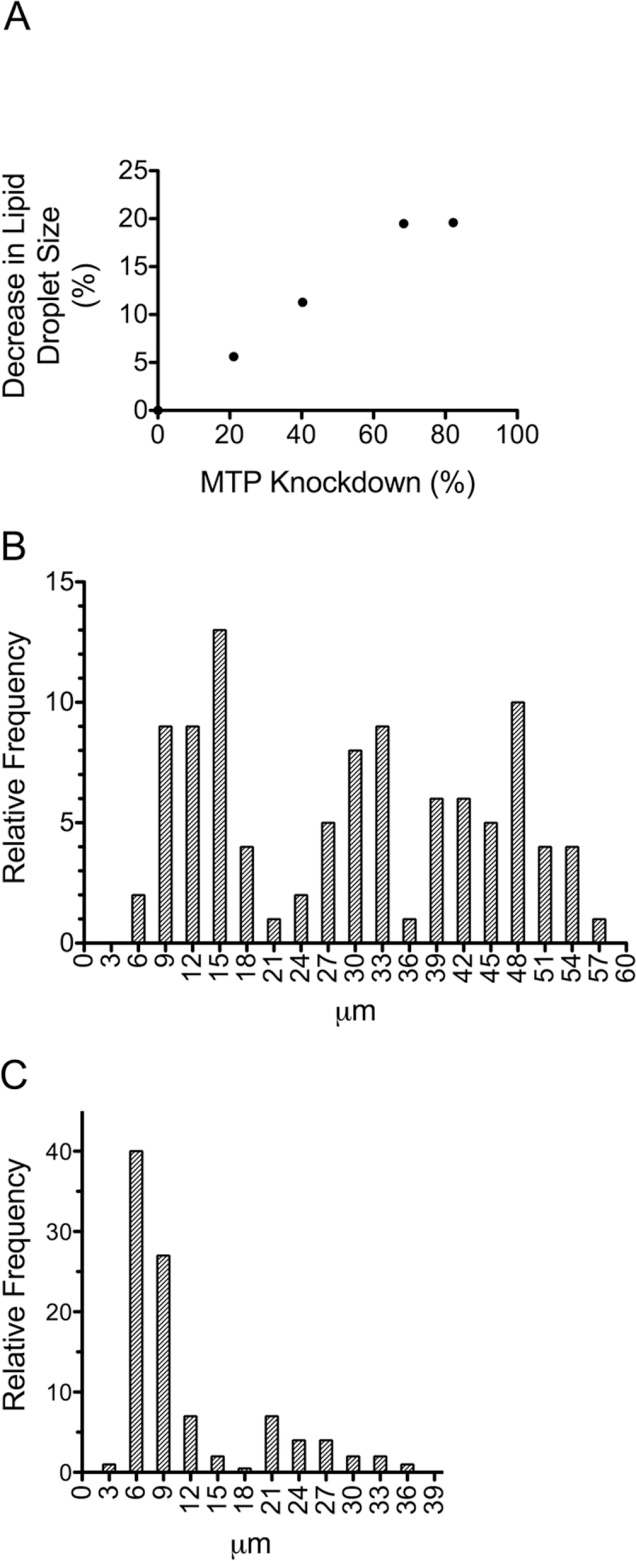
Effects of MTP knockdown on lipid droplet size and distribution. (A) Lipid droplet size in white fat from control and knockdown mice was determined as described in Fig 5. The decrease in lipid droplet size (%) in MTP knockdown mice compared with control was calculated and plotted versus MTP knockdown (%). (B,C) Size distribution of lipid droplets in brown fat from control (B) and knockdown (C) mice. Raw data from Fig 5 were used in this analysis.

We explored potential relationships between lipid droplet size, tissue mass, and percent MTP knockdown in white and brown fat. We found no relationship between the percent MTP knockdown and tissue mass or between lipid droplet size and tissue mass. However, we did find that the percent decrease in lipid droplet size in white fat was linearly related to the percent MTP knockdown, i.e., the more efficient the knockdown the greater the reduction in lipid droplet size (Fig 6A). Interestingly this relationship was true only up to ~70% knockdown. We did not observe any additional decrease in lipid droplet size with knockdowns greater than 70%. Similarly, knockdowns greater than 70% in brown fat produced comparable decreases in lipid droplet diameter. We did find that reduction in diameter of lipid droplets in brown fat was associated with a remarkable change in overall size distribution. For example, in brown fat from control mice we found a tri-modal distribution of sizes (Fig 6B), whereas in knockdown mice the distribution was shifted to a unimodal distribution of small droplets (Fig 6C). This is consistent with the hypothesis that MTP is involved in lipid droplet expansion.

**Fig 7:**
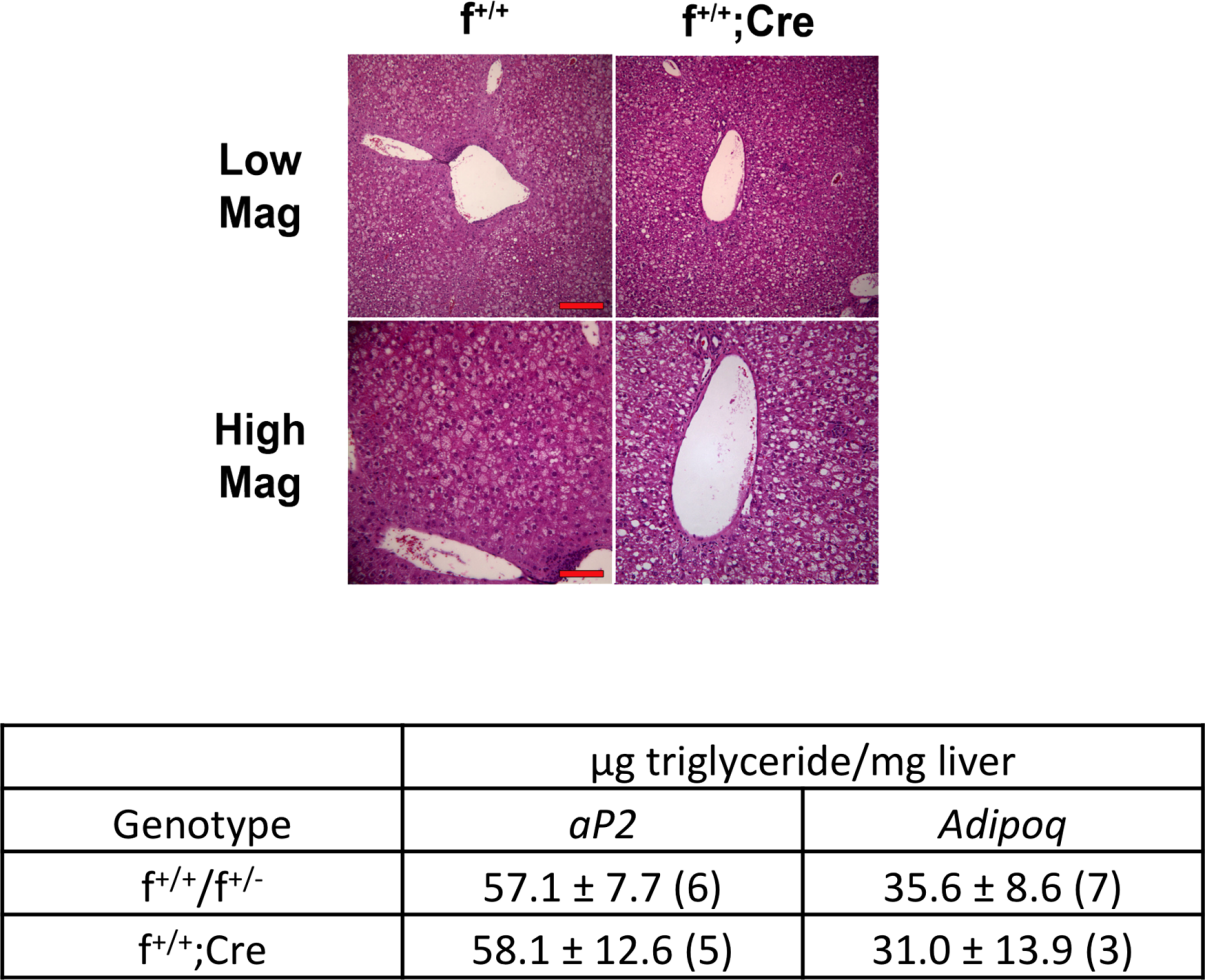
Fatty livers from f^+/+^ and f^+/+^;*Cre* mice on high fat diet. Top: Male mice derived from *Adipoq* crosses were fed regular rodent diet for 14 weeks at which time they were switched to the high fat diet for 8 weeks. Mice were sacrificed and pieces of liver fixed, sectioned and stained with hematoxylin and eosin for histochemical analyses. Images were captured at low (10X, bar = 200 μm) and high (20X, bar = 100 μm) magnifications. Bottom: Lipids were extracted from pieces of liver from control and knockdown mice, and triglycerides were analyzed by gas chromatographic methods. Data represent mean ± s.e.m.

We also examined livers from control and knockdown mice on the high-fat diet (Fig 7). Lower magnification (10X) images revealed lipid droplets in hepatocytes throughout the sections. Very few, if any, hepatocytes in control or knockdown livers did not contain lipid droplets. Higher magnification (20X) revealed cells filled with small lipid droplets. We extracted lipid from livers and quantitated the triglyceride content by gas chromatographic methods. There were no differences in the amount of triglyceride in the livers of control and knockdown mice from either the *aP2* or the *Adipoq* crosses (Fig 7).

## Discussion

MTP has long been known to be essential for the assembly of triglyceride-rich apolipoprotein (apo) B-containing lipoproteins by hepatocytes and enterocytes [10], presumably transporting neutral lipid from the membrane of the endoplasmic reticulum(ER) to nascent lipoproteins forming in the lumen of the ER. Once thought to be only present in the liver and intestine, studies have shown that MTP is expressed in a wide range of tissues, including myocardium [11–13], retina [14], kidney [15, 16], yolk sac [17, 18], and placenta [19]. MTP is also expressed in ovary and testis [15], as well as fat [1], tissues that do not express apoB nor do they secrete lipoprotein particles. Within these tissues MTP may serve specialized needs in lipid trafficking and/or storage beyond the classical role of triglyceride transport.

Our laboratory was the first to report the presence of MTP in adipocytes of mouse white and brown fat as well as in 3T3-L1 cells [20]. Given the known function of MTP as a lipid transfer protein, it was easy to develop a number of hypotheses as to the function of MTP within the adipocyte. An intriguing hypothesis for us was that MTP was in some way involved in lipid droplet formation and/or maturation. If this hypothesis was true, we postulated that MTP might regulate the amount of lipid accumulating within the cell. Extrapolating this to animals, we speculated that MTP might play a role in the accretion of cellular fat and thus influence body weight. To pursue this hypothesis we initiated studies using mice in which MTP expression in white and brown fat was suppressed. The mouse model was developed using a standard *Cre*-Lox approach. Mice in which exons 5 and 6 of the *Mttp* gene are flanked by *loxP* sites (f^+/+^) were crossed with mice expressing *Cre* recombinase under the control of either the *aP2* (*FABP4*) promoter or the *Adipoq* (adiponectin) promoter. The resulting mice (f^+/+^;*Cre*), generated from either *Cre*-recombinase, exhibited variable decreases in MTP expression in white and brown adipose tissue (Fig 1C). In white fat MTP expression was reduced from 20–90% with an average of approximately 55%. In a similar manner MTP expression in brown fat was reduced from 30–90% with an average reduction of 65% using *aP2-Cre* mice and 82% with *Adipoq-Cre* mice. Importantly, we never observed a mouse in which MTP expression in adipose tissue was completely deleted; hence we refer to the mice as knockdown mice. We were somewhat puzzled by the inability to obtain efficient and consistent knockdown of MTP within and between litters. We do not believe it is related to the *Cre* system as we observed variability in both *aP2*- and *Adipoq-Cre* crosses. One possible explanation could relate to the fact that adipose tissue contains a number of different cell types, some of which express MTP. MTP expression in these non-adipocytic cells could affect overall adipose tissue MTP expression. Macrophages are probably the most abundant non-adipocytic cell present. In lean mice they represent ~10% of the total cells in adipose tissue [21], and they do express MTP; however, macrophages also express *aP2*. Consequently MTP should be deleted in these cells in the *aP2-Cre* mice. Other non-adipocytic cells include fibroblasts and vascular endothelial cells as well as immune cells, such as mast cells, eosinophils, B cells and T cells. MTP is not expressed in fibroblasts or endothelial cells, but there is evidence that B cells express MTP [22]. However, our microscopic analysis of adipose tissue indicates that these cells make up only a very small percentage of the tissue mass and thus their contribution to total adipose tissue MTP levels would be minimal and could not account for the wide variability of knockdown seen in our studies. Consequently, we do not believe that the variability in knockdown observed within and between litters can be accounted for by the variety of cells present in adipose tissue.

Contrary to our initial hypothesis, we found no differences in weight between control (f^+/+^, f^+/-^) and knockdown (f^+/+^;*Cre*) mice from either the *aP2* or the *Adipoq* crosses when the animals were fed a regular rodent diet or a high fat diet (Figs 2 and 3). In addition, we found no differences between control and knockdown mice from *aP2* or *Adipoq* crosses with regard to the mass of white fat, brown fat, or liver (Table 1), body composition (Table 2), serum triglyceride, cholesterol or unesterified fatty acid levels (Table 3), or hepatic triglyceride content (Fig 7). However, what we did find was that the presence of MTP in adipocytes significantly influenced the size of lipid droplets in both brown and white fat. Adipocytes in white fat from knockdown mice were significantly smaller (5–25%) than adipocytes from control mice whether the animals were fed regular rodent or high fat diet (Figs 4 and 5). Furthermore, we found a linear relationship between the decrease in lipid droplet size and the percent MTP knockdown (Fig 6A), suggesting MTP plays a role in determining lipid droplet size. Why this relationship holds only for knockdowns up to 70% is unknown, but probably reflects the fact that maturation of lipid droplets is modulated by numerous factors. It might also suggest that the contribution of MTP to lipid droplet growth is limited to a certain time during maturation, a hypothesis that will be tested in future studies.

Lipid droplets in brown fat from knockdown mice on regular rodent diet were nearly 50% smaller than those found in brown fat from control mice (Figs 4 and 5). In addition, there was a dramatic shift in size distribution in the brown fat of the knockdown mice to smaller droplets (Fig 6B and 6C). Our results suggest that MTP is involved in the growth and maturation of lipid droplets; however, MTP apparently does not control overall fat accumulation as we found no differences in body weights, body composition, or mass of white fat depots between control and knockdown mice. Thus we conclude that to offset the significant decrease (40–60%) in adipocyte cell volume in the white fat of knockdown mice, there must be an increased number of cells.

Previous studies from our laboratory using 3T3-L1 cells have provided several lines of evidence for the involvement of MTP in lipid droplet formation/maturation. We have shown: 1) MTP expression increases in 3T3-L1 cells as the cells are induced to differentiate [1, 2]; 2) As 3T3-L1 cells differentiate MTP moves from the juxtanuclear region of the cell to the surface of the lipid droplet [2]; 3) MTP associates preferentially with small lipid droplets (<5 um) and is prominent but not limited to points of contact of lipid droplets [1]; and 4) Inhibition of MTP lipid transfer activity has no effect on the differentiation of 3T3-L1 cells as assessed by the percent of cells containing lipid droplets or the number of droplets/cell [2]. Our *in vivo* studies complement the cellular studies and provide the first direct evidence that MTP plays an important role in lipid droplet growth. The mechanism by which MTP regulates expansion is unknown. A number of models for lipid droplet expansion have been proposed. The models involve expansion via (1) fusion of small droplets to form larger droplets [23, 24], (2) synthesis of core triglyceride at the surface of the droplet [25–27], and (3) transfer of triglyceride from adjacent droplets with one droplet growing at the expense of another [28, 29]. Our observations from cellular studies that lipid transfer activity of MTP is not critical for droplet growth coupled with our microscopic studies showing MTP prominently located at points of contact of lipid droplets would favor the fusion model as a mechanism. In that regard Read *et al*. [30] proposed that during lipoprotein assembly in the endoplasmic reticulum, helix A within the MTP protein (amino acids 725–736) acts like a viral fusion peptide, disrupting the luminal phospholipid monolayer and providing access to neutral lipid synthesized in the ER bilayer. In a similar manner, disruption of the phospholipid monolayer on the surface of adjacent droplets could facilitate fusion.

In a recent report Bakillah and Hussain [3] reported that ablation of the *Mttp* gene in adipose tissue of mice using the *Cre*-lox approach with *aP2-Cre*-recombinase produced mice (A-*Mttp*^*-/-*^) that were resistant to high fat diet-induced obesity. In addition, they reported that A-*Mttp*^*-/-*^ had reduced adipose tissue with increased numbers of smaller size adipocytes and less macrophage infiltration compared with wild-type mice (*Mttp*^*fl/fl*^). They also reported that A-*Mttp*^*-/-*^ mice had moderate increases in plasma triglycerides and were protected from high-fat diet-induced fatty liver. Finally, they reported that adipose tissue of the A-*Mttp*^*-/-*^ mice had significantly lower PPARγ expression and its downstream targets, leading them to conclude that MTP modulates adipogenesis by influencing PPARγ expression, and therefore plays a role in the accretion of lipids to form larger lipid droplets. They also speculated that inhibition of adipose tissue MTP might be useful as an anti-obesity drug.

While our studies and those of Bakillah and Hussain [3] are in agreement that decreased expression of MTP in adipose tissue leads to smaller lipid droplets and decreased adipocyte size, our results do not support the conclusion that decreased expression of MTP in adipose tissue produces resistance to high fat diet-induced obesity or protection from high fat diet-induced fatty liver. In trying to understand the differences between the two studies, it is important to note that the diet in our studies contained 42% of kcal/g as fat compared with 60% kcal/g fat in the study of Bakillah and Hussain [3]. In addition, Bakillah and Hussain [3] began feeding the high fat diet at 8 weeks of age and fed for 15 or 24 weeks, whereas we began feeding the high fat diet at 14 weeks of age and fed for 8 weeks. In one study we fed the high fat diet for 24 weeks from weaning (4 wks). However, with the exception of the changes in plasma triglyceride levels, one would predict that our diet with its lower fat content would actually be more likely to produce the changes seen in their study.

In both studies the fat-specific MTP knockdown mice were developed using transgenic mice expression *Cre*-recombinase under the control of the *aP2* (*FABP4*) promoter and mice with *loxP* sites surround exons 5 and 6 of MTP. All known isoforms of MTP should be targeted by this approach. Although *aP2* was originally thought to be an adipocyte-specific protein, more recent studies have shown that it is also expressed in a number of other tissues and cell types including brain, macrophages, CNS and PNS, eye, heart, intestine, kidney, adrenal, ovary, testis, thymus, spleen, adipocyte precursors, and embryonic tissues [31, 32]. Many of these tissues express MTP. Thus, *Cre* under the *aP2* promoter may lead to secondary effects, which can be especially problematic in metabolic studies. Furthermore, Jeffery *et al*. [31] characterized *Cre* recombinase activity in adipocytes using the membrane-Tomato/membrane-GFP (mT/mG) dual fluorescent reporter and found that the *aP2*-*Cre* line does not label the majority of adipocytes in white adipose tissue depots. This may account for the modest reduction in MTP expression in this tissue. Because of our concerns with *aP2*-*Cre*–the seemingly inefficient targeting of adipocytes, the lack of tissue specificity and the potential for secondary effects–we switched to adiponectin-*Cre* (*Adipoq*) mice. *Adipoq*-*Cre* mouse lines have been shown to have a high specificity for adipose tissue in both brown and white fat [31, 33]. Further, Lee *et al*. showed that *Adipoq*-*Cre* provided more efficient recombination than *aP2*-*Cre* [33]. We found slightly greater knockdown of MTP in white and brown fat using the *Adipoq*-*Cre* mice; however, the overall results from the *Adipoq*-*Cre* mice were identical to those from the *aP2*-*Cre* transgene, which, we believe, is a strong validation of our results.

A major issue in both studies is the actual effectiveness of the knockdown. Our immunblot analyses of tissue MTP levels demonstrated a striking variability of the knockdown between litters and even within litters. The overall average knockdown using either transgene was approximately 50%. Bakillah and Hussain do not provide any direct measurements of MTP protein, such as immunoblotting; however, they state that A-*Mttp*^-/-^ mice have a “significant MTP deficiency in adipose tissue” based on their finding of a >50% reduction in MTP activity in inguinal fat pads. While a 50% reduction in MTP protein/activity might represent a statistically significant decrease, we have no information on how much MTP is actually needed to maintain its normal function in adipocytes. In this regard it is important to note that most individuals who are obligate heterozygous for abetalipoproteinemia have no symptoms and no evidence of reduced plasma lipid levels in spite of a 50% reduction in MTP activity, presumably in all tissues that express the protein [34, 35]. Thus, a 50% reduction might not be sufficient to produce a consistent phenotype. Furthermore, given the variability in the knockdowns in white adipose tissue (Fig 1), it is possible that the phenotype is also variable, although we have been unable to identify any unique differences between control and knockdown mice when the data are analyzed with respect to the degree of knockdown.

In conclusion, our studies demonstrate that MTP is involved in the expansion of lipid droplets in both brown and white fat of mice. Reduction of MTP expression in white adipose tissue leads to a 40–60% decrease in adipocyte volume and up to a 90% reduction in volume of lipid droplets in brown fat. Interestingly, this reduction in volume of adipocytes and lipid droplets had no effect on body composition or overall accumulation of body fat whether animals were fed a regular rodent diet or a high fat diet. Whereas the precise mechanism by which MTP regulates droplet size is unclear, our results combined with previous cellular studies support a fusion model of expansion. Future studies will explore the precise mechanism by which MTP controls droplet volume.
